# FERMT2 drives anoikis resistance and peritoneal metastasis by enhancing extracellular matrix deposition in gastric cancer

**DOI:** 10.1007/s10120-025-01602-0

**Published:** 2025-03-01

**Authors:** Chao He, Zheng Zhou, Yan Yang, Songting Zhu, Haiyong Wang, Lisong Teng

**Affiliations:** Department of Surgical Oncology, The First Affiliated Hospital, Zhejiang University School of Medicine, Hangzhou, China

**Keywords:** Gastric cancer, Anoikis resistance, Metastasis, FERMT2

## Abstract

Peritoneal metastasis is a critical step in the progression of gastric cancer (GC), yet its underlying mechanisms remain poorly understood. Here, we identify FERMT2, a member of the Kindlin protein family, as a key regulator of anoikis resistance (AR) and peritoneal metastasis in GC. FERMT2 expression increases in a suspension-time-dependent manner and is associated with higher pathological grade, advanced clinical stage, and poorer prognosis. Functional studies in vitro and in vivo demonstrate that FERMT2 promotes AR and facilitates peritoneal metastasis. Mechanistically, FERMT2 suppresses the ubiquitination of SOX2, thereby enhancing its stability and up-regulating FN1 transcription. Furthermore, we report that TGFβ-RI expression also increases in a suspension-time-dependent manner, forming a positive feedback loop with FERMT2 via TGFβ-1/TGFβ-RI signaling. This feedback loop drives extracellular fibronectin matrix deposition, strengthens cell–matrix interactions, and supports AR. These findings establish FERMT2 as a pivotal mediator of peritoneal metastasis in GC, offering insights into its potential as a therapeutic target.

## Introduction

According to 2022 statistics from the International Agency for Research on Cancer, nearly 20 million new cancer cases were reported globally, with cancer-related deaths reaching 9.7 million. Gastric cancer ranks as the fifth most common malignancy worldwide and remains a leading cause of cancer-related mortality [1]. A major factor contributing to the poor survival rate of GC is late-stage diagnosis, which significantly increases the risk of both local and systemic metastases. Peritoneal metastasis is a common progression of GC, often occurring after radical gastrectomy and associated with a poor prognosis, with a median survival of less than six months [2]. Despite its clinical significance, the underlying mechanisms driving GC peritoneal dissemination remain poorly understood.

Anoikis, a programmed cell death triggered by the loss of cell–cell and cell-extracellular matrix (ECM) adhesion, serves as a critical barrier against metastatic dissemination. It is primarily mediated through mitochondrial dysfunction or activation of apoptotic receptors on the cell surface. While anoikis helps maintain tissue homeostasis by eliminating mislocalized or detached cells, cancer cells can evade anoikis, allowing them to survive in unfavorable conditions and contribute to cancer progression and metastasis [3, 4]. The establishment of peritoneal metastases requires GC cells to detach from the primary tumor, overcome anoikis, and colonize distant sites within the abdominal cavity [5]. Therefore, understanding the molecular mechanisms underlying AR in GC may provide key insights into the pathogenesis of peritoneal dissemination.

Kindlin-2 (FERMT2), a member of the Kindlin protein family, is widely expressed across various tissues [6] and plays a central role in integrin activation, cell-ECM adhesion, and migration [7]. In GC, FERMT2 upregulation has been linked to increased invasive potential via tumor-associated macrophages [8] and has been implicated in lymphatic metastasis [9]. Notably, elevated FERMT2 expression correlates with poor clinical outcomes across multiple solid tumors, including GC [10].

Despite these findings, the role of FERMT2 in peritoneal metastasis remains unclear. FERMT2 has been implicated in regulating podocyte-matrix adhesion, fibronectin matrix deposition, and integrin activation [11]. Cancer cells that detach from the ECM frequently enhance fibronectin deposition and activate integrins to reinforce cell–cell junctions—critical processes for acquiring AR [12]. Based on these insights, this study aims to elucidate the role of FERMT2 in regulating AR in GC cells, shedding light on its potential as a therapeutic target.

## Materials and methods

### Cell culture and reagents

Four human GC cell lines (HGC-27, AGS, MKN45, and SNU-1) were obtained from the Cell Bank of the Chinese Academy of Sciences (Shanghai, China). The cells were cultured in RPMI-1640 medium (Gibco) supplemented with 10% fetal bovine serum (FBS) at 37 °C in a humidified incubator with 5% CO2. Cancer-associated fibroblasts (CAFs) were isolated from fresh GC tissues and immortalized after transformation. CAFs were maintained in DMEM (Gibco) with 10% FBS under identical culture conditions. The primary antibodies used in this study are listed in Table 1.

**Table 1:** The primary antibodies applied in this study were shown as follows

Name	Article numbers (brand)
FERMT2	11,453–1-AP (Proteintech)
SOX2	#23,064 (CST)
Fibronectin	66,042–1-Ig (Proteintech)
β-actin	81,115–1-RR (Proteintech)
cleaved caspase-3	AF7022 (Affinity Biosciences)
cleaved PARP	#5625 (CST)
TGFβ-RI	30,117–1-AP (Proteintech)
Integrin-α5	10,569–1-AP (Proteintech)
Integrin-β1	26,918–1-AP (Proteintech)
Phalloidin	PF00001 (proteintech)
secondary antibodies	AS014 (Abclonal), SA00001-1 (Proteintech)
Fluorescent secondary antibody	SA00013-4, SA00014-10 (Proteintech)

### Tissue microarray sample collection

A total of 143 GC tissue samples were collected from patients who underwent surgical resection at the First Affiliated Hospital of Zhejiang University between January and December 2016. These samples were used to construct a tissue microarray (TMA) for immunohistochemical (IHC) analysis. Clinicopathological data and follow-up survival information were obtained, including assessments of peritoneal metastasis based on surgical records, pathology reports, and postoperative evaluations (e.g., abdominal CT, MRI, PET-CT, and biopsy results). Patients were informed about the use of their samples for scientific research and the requirement for informed consent was waived by the Clinical Research Ethics Committee of the First Affiliated Hospital of Zhejiang University (Approval No: 2024–0956).

### Cell transfection

The FERMT2 and SOX2 lentiviral expression vectors were constructed by cloning the FERMT2 cDNA fragment (NM_006832.3) and SOX2 cDNA fragment (NM_003106.4), respectively, into shuttle vectors. For gene silencing, specific short hairpin RNAs (shRNAs) targeting FERMT2 and small interfering RNAs (siRNAs) targeting SOX2 were designed (sequences provided in Table 2). Recombinant lentiviral vectors were packaged and purified by Genomeditech (Shanghai, China). GC cells were subsequently infected with the lentiviral vectors and subjected to selection with puromycin or blasticidin for 2 weeks.

**Table 2:** Primer, siRNA and shRNA sequences in this study were shown as follows

Name	Sequence (5ʹ–3ʹ)
FERMT2 forward	CCTGATTCCAGTTGCAGAAGGC
FERMT2 reverse	CATGGTCTTGCCTTTGGAGGCT
FN1 forward	GAGGGCAGAAGAGACAACATGAA
FN1 reverse	CCCTTCATTGGTTGTGCAGATTT
SOX2 forward	GACTTCACATGTCCCAGCACTAC
SOX2 reverse	ATTTGCTGTGGGTGATGGGATTT
GAPDH forward	GGAGCGAGATCCCTCCAAAAT
GAPDH reverse	GGCTGTTGTCATACTTCTCATGG
SOX2-FN1 CHIP Primer –forward	GGAAACTGATATTTGCTGGGTGT
SOX2-FN1 CHIP Primer –reverse	ACCTTCAGTAATTGCCACAGGA
sh-FERMT2-1	AGCAGATCACGACTGATATAA
sh-FERMT2-2	GGTGGAGAAACTCGATGTAAA
sh-FERMT2-3	CCGAAGAACTTTCTCTCTTAA
sh-Control	TTCTCCGAACGTGTCACGT
si-Sox2-1	CUGCAGUACAACUCCAUGA
si-Sox2-2	GGAGAAAUUUUCAAAGAAA
si-Control	UUCUCCGAACGUGUCACGU

### Induction of anoikis

GC cells were resuspended at a density of 1 × 10^5 cells/ml in RPMI-1640 medium with 10% fetal bovine serum and plated (2 ml/well) onto ultra-low attachment 6-well plates (Corning, #3471). At specified suspension intervals, cells were collected for protein extraction or viability analysis.

### Cell migration and invasion assays

Cell migration and invasion were assessed using 24-well Transwell chambers with 8.0-μm pore polycarbonate filters (Corning, #3422). For migration assays, 5 × 10^4 cells were seeded into uncoated upper chambers, while 1 × 10^5 cells were used for invasion assays in Matrigel-coated chambers (Corning, #354,234). The upper chambers contained 200 µL of serum-free medium, and the lower chambers 500 µL of complete medium. After 24 h, cells on the lower filter surface were fixed with 4% paraformaldehyde, stained with 0.05% crystal violet, and washed with distilled water. Non-migrated cells were removed using a cotton swab. Migrated cells were counted in three random fields per sample at 20 × magnification under a light microscope.

### Soft agar colony formation assay

Prepare 1.2% and 0.7% agar solutions and keep them in liquid form at 37 °C after autoclaving. For the bottom layer, mix 1.2% agar with 20% RPMI-1640 medium (1:1), add 2 mL per well of a 6-well plate, and allow it to solidify at room temperature. For the top layer, mix 0.7% agar with 20% RPMI-1640 medium (1:1), incorporate 10^3 cells, and overlay this mixture onto the solidified bottom layer. After the top layer solidifies, incubate the plate at 37 °C for 2–3 weeks, adding 200 µL of medium to each well every three days to maintain culture conditions.

### Western blotting

Total proteins were extracted from cultured cells using RIPA buffer (Beyotime Inc., China) with phosphatase and protease inhibitors (1:100 dilution). The lysates were centrifuged at 12,000 × g for 20 min at 4 °C, and the supernatant was collected. Equal protein amounts (20 μg) were loaded per lane, separated by SDS-PAGE, and transferred to PVDF membranes (Millipore, USA). Membranes were incubated overnight at 4 °C with primary antibodies, followed by incubation with an HRP-conjugated secondary antibody for 1 h at room temperature. β-actin was used as a loading control. Protein bands were visualized using the ECL Chemiluminescence Detection Kit (Yeasen Biotechnology, China). Primary antibodies are listed in Table 1.

### RNA extraction and RT-qPCR analysis

The RNA Purification Kit (EZBioscience, B0004DP) was used to extract RNA from cells. The RT Reagent Kit (AG) was applied for reverse transcription using 1 μg of RNA in a total reaction volume of 20 μL. Universal SYBR Green Fast qPCR Mix (ABclonal) was used for qPCR on the CFX96 Real-Time PCR Detection System (StepOnePlus). The relative expression levels of each gene were normalized to GAPDH expression, and each experiment was repeated at least three times.

### Immunohistochemistry

Paraffin-embedded sections were baked at 65 °C for 2 h for tissue fixation. They were then deparaffinized with xylene, dehydrated through graded alcohols, and subjected to antigen retrieval in sodium citrate buffer. The primary antibody was applied based on tissue size and incubated at 37 °C for 60 min. Afterward, an enzyme-labeled goat anti-rabbit IgG polymer was added and incubated at 37 °C for 20 min. DAB chromogenic solution was applied for 2 min at room temperature. Sections were counterstained with hematoxylin for 20 s, rinsed with tap water for bluing, air-dried, and coverslipped with neutral resin.

### Immunoprecipitations

Whole-cell protein from GC cells cultured in a 10 cm dish was extracted using 1 mL of RIPA buffer (Sigma). The extract was incubated overnight at 4 °C with BeyoMag™ Protein A + G Magnetic Beads (Beyotime) and the corresponding antibody on a vertical rotator. The precipitates were collected using a magnetic rack. After adding 1 × loading buffer, the samples were heated at 100 °C for 15 min. Co-precipitated proteins were analyzed by Western blotting.

### Chromatin immunoprecipitation (ChIP)

The BeyoChIP™ Enzymatic ChIP Assay Kit (Protein A/G Beads, P2083S) was used for chromatin immunoprecipitation. Micrococcal Nuclease (MNase) digestion was employed for chromatin fragmentation, replacing the traditional sonication method. *SOX2* antibody and normal Rabbit IgG (negative control) were used to precipitate chromatin fragments bound to the target protein. After column purification, the precipitated chromatin fragments were analyzed using methods including PCR followed by agarose gel electrophoresis and RT-qPCR. All experimental procedures were performed according to the manufacturer’s protocol.

### Immunofluorescence

GC cells (1 × 10^4) were seeded in confocal dishes and fixed with 4% paraformaldehyde for 15 min. After permeabilization with 0.5% Triton X-100 in PBS for 20 min, cells were blocked with 5% BSA for 30 min. Without washing off the blocking solution, cells were incubated overnight at 4 °C with primary antibody (1:200 in 1% BSA). Afterward, cells were incubated with fluorescent secondary antibody (1:200 in 1% BSA) for 1 h at room temperature, protected from light. DAPI was used for nuclear staining for 5 min. Samples were mounted with an anti-fade medium and imaged using a Leica-S5 confocal microscope.

### In vivo* assays for tumor anoikis*

Four-week-old female nude mice were purchased and maintained according to ethical guidelines approved by the Clinical Research Ethics Committee of the First Affiliated Hospital of Zhejiang University (Approval No. 2024–1299). In this experiment, mice were randomly assigned to experimental groups using the computer-generated randomization method from the “randomizeR” package (version 3.0.2) to ensure unbiased allocation. The researcher administering treatments and assessing outcomes was blinded to the group assignments to minimize bias in data analysis and interpretation. For the peritoneal metastasis model, 5 × 10^6 cells in 200 µL of PBS were injected into the peritoneal cavity of mice. The TGF-β receptor kinase inhibitor SB-431542 (10 μM, diluted in 1 µL of DMSO) was administered via intraperitoneal injection on the 1 ^st^, 3 ^rd^, 5th and 7 ^th^ days following tumor cell inoculation in 200 µL of PBS. After 4 weeks, mice were sacrificed, and the peritoneal cavities were examined. Tumor nodules were carefully dissected, pooled, photographed, and weighed.

### Acquisition and processing of single-cell sequencing data

Single-cell sequencing data from primary GC and peritoneal metastasis lesions were retrieved from the GEO database (GSE210347 and GSE163558). Batch effects across samples were corrected using the "Harmony" R package. Cell and gene filtering, as well as normalization and scaling, followed protocols described in a previous study[13]. Cell subpopulations were identified using the "FindClusters" function in the "Seurat" R package, with a default resolution of 0.5, and renamed based on canonical markers. FERMT2 expression levels were extracted from the gene expression matrix and compared between primary and peritoneal metastasis lesions.

### Statistical analysis

All experiments were performed independently at least three times, and all data are expressed as “mean ± SD.” For comparisons between two groups, we used Student’s *t*-test for normally distributed quantitative data and the nonparametric Mann–Whitney U test for quantitative data that did not follow a normal distribution. Statistical analyses were conducted using R (version 4.3.1), with P < 0.05 considered statistically significant.

## Result

### FERMT2 upregulation enhances fibronectin secretion and promotes anoikis resistance in gastric cancer cells

**Fig. 1:**
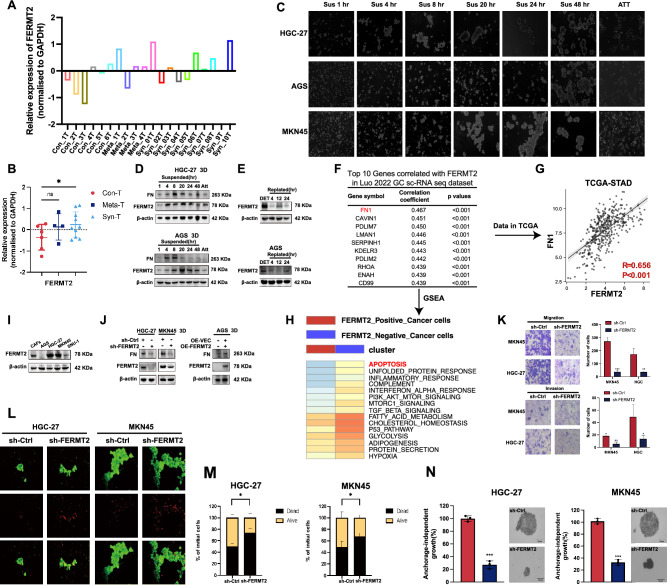
FERMT2 up-regulation in detached conditions promotes anoikis resistance in GC cells. **A, B** The relative FERMT2 protein expression in primary tumors with or without peritoneal metastasis was compared. **C** HGC-27, AGS, and MKN45 GC cells were cultured under attachment or suspension conditions for the indicated times, and representative images were taken. **D** Immunoblot analysis of fibronectin and FERMT2 expression in HGC-27 and AGS cells under attachment (ATT) and detachment (DET) conditions at different time points. **E** After 24 h of detachment, GC cells were replated on ECM and analyzed by immunoblotting. **F, G** Correlation analysis between fibronectin and FERMT2 mRNA levels in single-cell RNA-seq data from Luo et al. and in TCGA gastric cancer (STAD) dataset. **H** Gene set enrichment analysis (GSEA) comparing gastric cancer cells with high and low FERMT2 expression. **I** FERMT2 and fibronectin expression levels in four GC cell lines and GC-associated fibroblasts. **J** shRNA targeting FERMT2 was introduced into HGC-27 and MKN45 cells, and OE-FERMT2 plasmids were transfected into AGS cells, followed by 24-h suspension culture and immunoblot analysis. **K** Migration and invasion abilities of HGC-27 and MKN45 cells were compared between sh-Ctrl and sh-FERMT2 groups. **L, M** Calcein/PI staining and Trypan blue exclusion were used to assess the survival of suspended GC cells after 48 h of ECM detachment. **N** Colony size in soft agar assays was measured in HGC-27 and MKN45 cells after FERMT2 silencing. Scale bar = 20 μm. (^ns^*p* > 0.05; *p < 0.05; ***p < 0.001)

We conducted a quantitative, untargeted proteomic analysis on primary GC samples from patients with (synchronous, *n* = 10; metachronous, *n* = 4) and without (*n* = 6) peritoneal metastasis. Elevated FERMT2 expression was observed in primary tumors of patients with peritoneal metastasis (Fig. 1A, B) , suggesting its role in metastasis development. Previous studies showed that FERMT2 depletion in podocytes reduces integrin activation, matrix adhesion, and fibronectin deposition [11], all of which are critical for cell survival under detachment conditions [12, 14]. We hypothesize that FERMT2 regulates AR by enhancing fibronectin secretion and promoting cell aggregation.

Upon detachment from the ECM, GC cells formed aggregates in a suspension-time-dependent manner (Fig. 1C). Both FERMT2 and fibronectin expression increased in HGC-27 and AGS cells under detachment, indicating FERMT2’s role in promoting AR through extracellular matrix deposition (Fig. 1D). After 24 h of detachment, FERMT2 expression decreased upon replating cells onto ECM (Fig. 1E). Single-cell sequencing (Luo et al., [15]) and TCGA-STAD data showed a positive correlation between FERMT2 and fibronectin mRNA levels (Fig. 1F, G). Gene set enrichment analysis revealed significant apoptosis pathway enrichment in FERMT2-negative GC cells (Fig. 1H), suggesting that FERMT2 depletion activates apoptosis.

FERMT2 expression was evaluated in four GC cell lines and GC-associated fibroblasts (a key source of extracellular fibronectin; Fig. 1I). After FERMT2 knockdown in HGC-27 and MKN45 cells, fibronectin synthesis decreased, while lentiviral-mediated FERMT2 overexpression increased fibronectin levels (Fig. 1J). Trypan blue and Calcein/PI staining showed that FERMT2 knockdown significantly reduced live cell numbers after 48 h of ECM detachment (Fig. 1L, M). Additionally, FERMT2 downregulation reduced colony size in soft agar (Fig. 1N) and inhibited cell invasion and migration (Fig. 1K).

### Upregulation of FERMT2 is associated with poor prognosis and enhanced peritoneal metastasis in GC

**Fig. 2:**
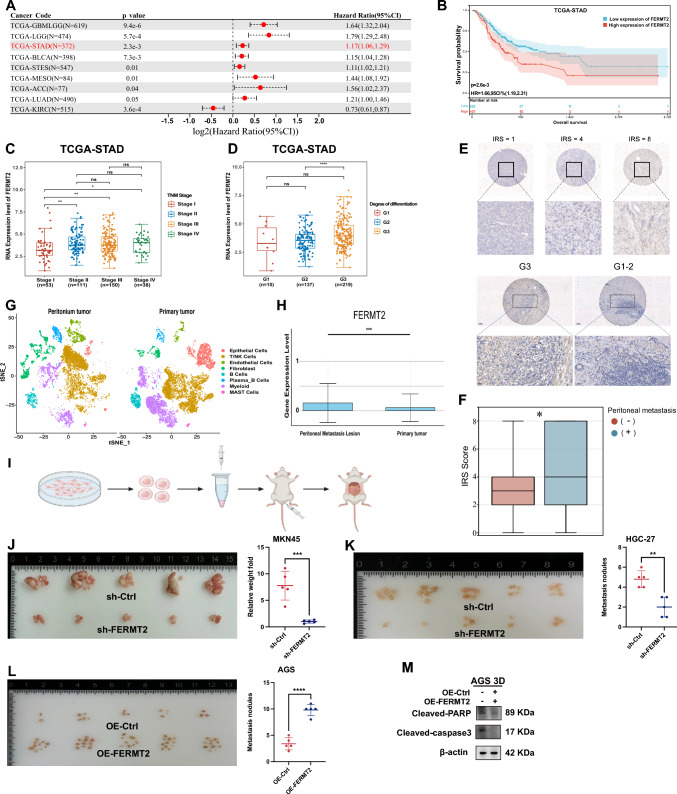
Upregulation of FERMT2 correlates with poor prognosis and enhanced peritoneal metastasis in GC cells. **A** Hazard ratio for elevated FERMT2 RNA expression across various cancer types with statistical significance. **B** Kaplan–Meier survival curve comparing GC patient outcomes with high versus low FERMT2 RNA expression in primary tumors. **C, D** FERMT2 RNA expression levels in relation to TNM stage and tumor differentiation in the TCGA-STAD dataset. **E** Representative IHC images of GC tissues, categorized by IRS score and differentiation degree. **F** IRS scores of primary GC lesions with or without synchronous or metachronous peritoneal metastasis. **G** t-SNE plot showing distinct cell subpopulations in primary GC lesions and peritoneal metastasis lesions. **H** Comparison of FERMT2 expression levels in cancer cells from primary and peritoneal metastasis lesions. **I** Schematic diagram illustrating the process of injecting GC cells into the abdominal cavity. **J, K, L** Effects of FERMT2 knockdown on peritoneal metastasis in HGC-27 and MKN45 cells, and the effect of FERMT2 overexpression in AGS cells, with comparisons of metastasis weight and number between the two groups. **M** Comparison of apoptosis marker expression in OE-Ctrl and OE-FERMT2 AGS cells following 48 h of suspension. Scale bar = 100 μm. (^ns^*p* > 0.05; *p < 0.05; **p < 0.01; ***p < 0.001; ****p < 0.0001)

In various cancer types, including gastric cancer (GC), higher FERMT2 RNA expression was linked to poorer prognosis and increased hazard ratios (P = 2.3e-3) (Fig. 2A). TCGA-STAD data showed that GC patients with high FERMT2 expression in primary tumors had worse survival outcomes (Fig. 2B). FERMT2 expression levels increased with higher TNM stages, and patients with poorly differentiated tumors had higher FERMT2 RNA expression (Fig. 2C, D). IHC of GC TMAs revealed that poorly differentiated tumors had higher IRS scores (Fig. 2E), and primary tumors with synchronous or metachronous peritoneal metastasis showed higher IRS scores than those without metastasis (Fig. 2F). The proportions of distinct cell subpopulations were different between primary tumors and peritoneal metastases (Fig. 2G), with FERMT2 RNA levels higher in peritoneal metastasis cells than in primary tumor cells (Fig. 2H).

A mouse GC peritoneal metastasis model was established (Fig. 2I). Stable FERMT2 knockdown in MKN45 and HGC-27 cells reduced peritoneal metastasis, while FERMT2 overexpression in AGS cells promoted peritoneal tumor formation (Fig. 2J, K, L). Under suspension conditions, FERMT2 overexpression in AGS cells inhibited apoptosis markers (cleaved-PARP and cleaved-caspase-3), indicating AR (Fig. 2M).

### FERMT2 interacts with SOX2 to enhance SOX2 protein stability

Previous research identified FERMT2 as a regulator of GC cell stemness, influencing suspension sphere formation and stemness markers, including SOX2[13]. Overexpression of SOX2 has been shown to increase fibronectin (FN1) expression in Schwann cells [16], leading us to hypothesize that FERMT2 regulates fibronectin secretion in GC cells by modulating SOX2 expression.

**Fig. 3:**
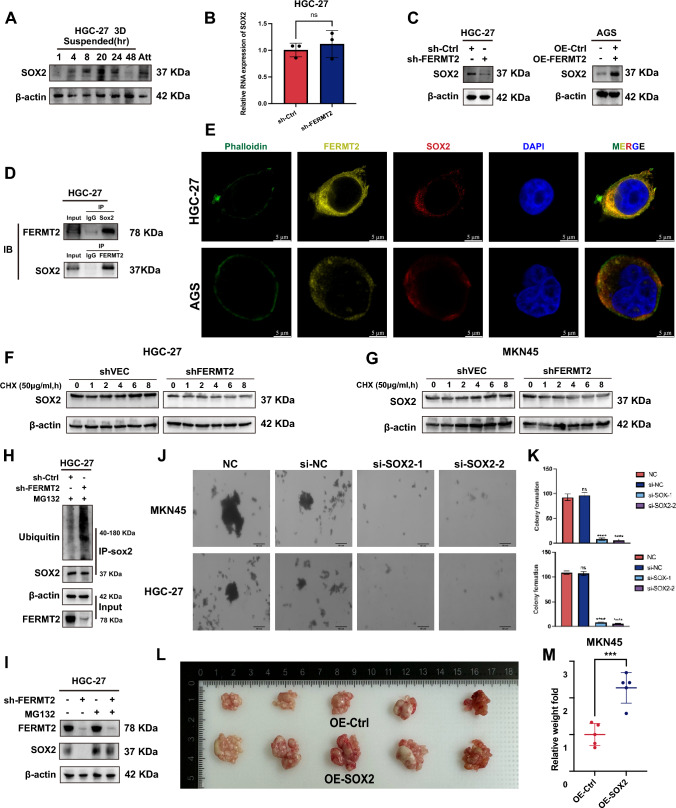
FERMT2 interacts with SOX2 to enhance SOX2 protein stability. **A** SOX2 protein expression in HGC-27 cells under attachment and suspension conditions at specific time points. **B** Comparison of SOX2 RNA expression in HGC-27 cells with sh-Ctrl and sh-FERMT2. **C** SOX2 protein expression in HGC-27 cells after FERMT2 knockdown and in AGS cells with FERMT2 overexpression. **D** Co-IP assays showing the interaction between FERMT2 and SOX2 in HGC-27 cells. **E** IF assays showing colocalization of FERMT2 and SOX2 in HGC-27 and AGS cells. **F, G** Cycloheximide-based protein stability assay for SOX2 in FERMT2-knockdown and control HGC-27 and MKN45 cells. **H** Ubiquitination levels of SOX2 after FERMT2 knockdown in HGC-27 cells. **I** Effect of proteasome inhibitor MG132 on SOX2 protein stability after FERMT2 knockdown. **J, K** Colony morphology and number of colonies in soft agar after SOX2 silencing in HGC-27 and MKN45 cells. **L, M** Peritoneal metastasis nodules from mice injected with OE-SOX2 or control lentivirus-transfected MKN45 cells, showing nodule weight and relative weight comparison. Scale bar = 50 μm. (^ns^*p* > 0.05; ***p < 0.001)

In HGC-27 cells, SOX2 expression increased in a suspension-time-dependent manner, mirroring the pattern of FERMT2 expression (Figs. 3A, 1D). Knockdown of FERMT2 did not affect SOX2 RNA levels (Fig. 3B) but reduced SOX2 protein levels. Conversely, FERMT2 overexpression in AGS cells increased SOX2 expression (Fig. 3C). Immunoprecipitation assays confirmed the interaction between FERMT2 and SOX2 (Fig. 3D), and immunofluorescence staining showed significant co-localization of both proteins in HGC-27 and AGS cells (Fig. 3E).

Knockdown of FERMT2 reduced SOX2 protein stability in HGC-27 and MKN45 cells (Fig. 3F, G), with increased ubiquitination of SOX2 in sh-FERMT2 cells compared to controls (Fig. 3H). Treatment with the proteasome inhibitor MG132 reversed this instability, reducing SOX2 ubiquitination and degradation (Fig. 3I). Furthermore, FERMT2 downregulation reduced colony formation in soft agar in both HGC-27 and MKN45 cells (Fig. 3J, K). Overexpression of SOX2 in MKN45 cells significantly promoted peritoneal tumor formation (Fig. 3L, M).

### SOX2 upregulated Fibronectin expression in GC

**Fig. 4:**
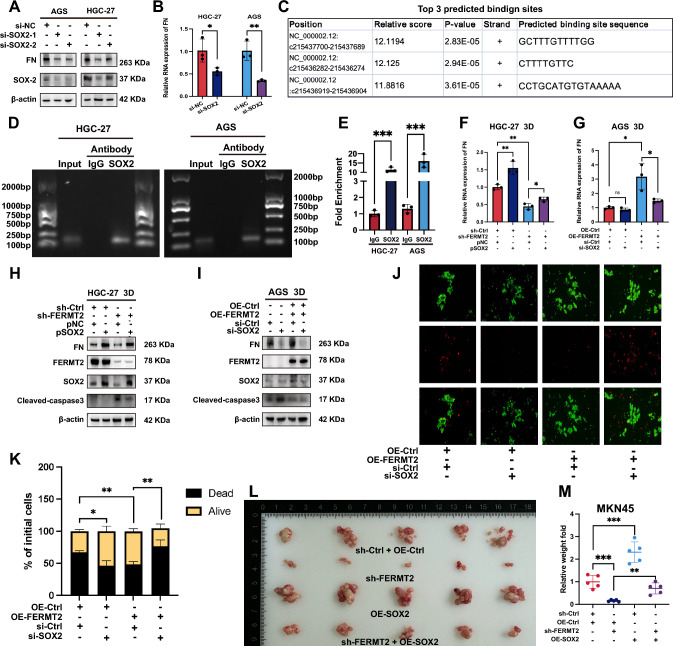
SOX2 transcriptionally upregulates Fibronectin in GC cells. **A, B** FN protein and RNA expression in AGS and HGC-27 cells transfected with SOX2-targeting siRNA. **C** Predicted SOX2 binding sites in the FN1 promoter, identified using human TFDB. **D, E** ChIP-PCR and RT-QPCR analysis showing SOX2 binding to the FN1 promoter region. **F** FN RNA expression in sh-Ctrl and sh-FERMT2 HGC-27 cells, after SOX2 plasmid transfection and 24-h ECM detachment. **G** FN RNA expression in OE-Ctrl and OE-FERMT2 AGS cells, after SOX2 siRNA transfection and 24-h ECM detachment. **H** FN and cleaved caspase-3 protein levels in sh-Ctrl and sh-FERMT2 HGC-27 cells, after SOX2 plasmid transfection and 24-h ECM detachment. **I** FN and cleaved caspase-3 protein levels in OE-Ctrl and OE-FERMT2 AGS cells, after SOX2-targeting siRNA transfection and 24-h ECM detachment. **J, K** Cell survival assessed by Calcein/PI and Trypan blue staining in suspended OE-Ctrl and OE-FERMT2 AGS cells, transfected with SOX2-targeting siRNA and 24-h ECM detachment. **L, M** Peritoneal metastasis of MKN45 cells transfected with sh-FERMT2, OE-SOX2 lentivirus, or both, showing nodule weight and relative weight comparisons (*p < 0.05; **p < 0.01; ***p < 0.001)

In AGS and HGC-27 cells, SOX2 knockdown reduced both protein and RNA levels of fibronectin (Fig. 4A, B). Top three predicted SOX2 binding sites on the FN1 promoter were identified using the human TFDB (Fig. 4C). ChIP-PCR and ChIP-RT-qPCR confirmed that SOX2 directly binds to the FN1 promoter in both cell lines (Fig. 4D, E).

SOX2 plasmid transfection partially reversed the downregulation of FN1 expression in sh-FERMT2 HGC-27 cells cultured in suspension for 24 h (Fig. 4F, H), while SOX2 siRNA transfection partially reversed FN1 upregulation in OE-FERMT2 AGS cells under the same conditions (Fig. 4G, I).

Calcein/PI staining and trypan blue assays showed that si-SOX2 transfection countered the increased cell viability induced by FERMT2 overexpression in AGS cells in suspension (Fig. 4J, K). Additionally, SOX2 overexpression partially rescued the peritoneal metastasis inhibition in MKN45 cells after FERMT2 knockdown (Fig. 4L, M).

In conclusion, SOX2 upregulates FN1 transcription, enhances ECM deposition and AR, and promotes peritoneal metastasis in GC.

### Role of positive feedback between TGFβ-1/TGFβ-RI signaling and FERMT2 in regulating anoikis resistance

SOX2, a pluripotency marker regulated by SMAD1 and SMAD3 downstream of BMPs and TGF-β/activin, plays a key role in acquiring AR. BMPs promote anoikis and inhibit metastasis by downregulating SOX2, while TGF-β/activin enhances cancer cell survival and metastasis via SOX2 upregulation [17]. TGF-β1 signaling is elevated in anoikis-resistant breast cancer cells [18], and FERMT2 knockdown reduces TβRI expression [19], suggesting FERMT2 may regulate AR through the TGF-β1 pathway. Based on these findings, we further investigate the role of TGF-β1 as upstream signaling in regulating SOX2 and FERMT2 in this study.

**Fig. 5:**
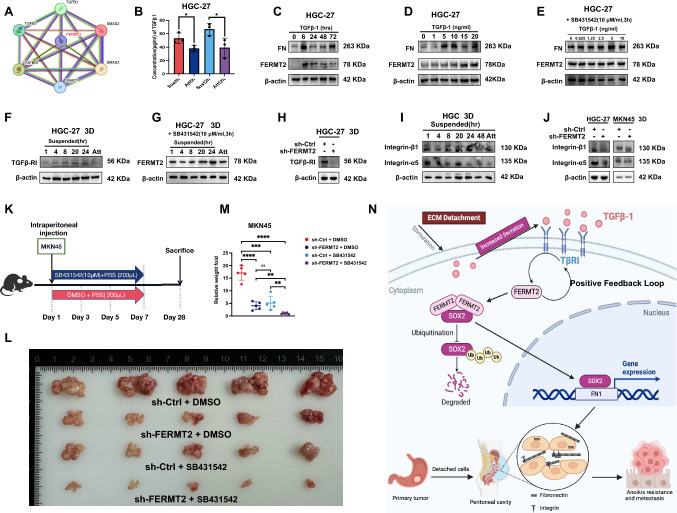
TGFβ-1/TGFβ-RI signaling and FERMT2 form a positive feedback loop regulating anoikis resistance. **A** Network diagram of FERMT2-related genes from the STRING database. **B** TGF-β1 concentration in cell supernatants under suspension conditions at different time points. **C** Expression levels of FEMRT2 and FN in HGC-27 cells treated with exogenous TGF-β1 (5 ng/ml) for various durations. **D** Expression levels of FEMRT2 and FN in HGC-27 cells treated with different concentrations of TGF-β1 for 24 h. **E** Expression levels of FEMRT2 and FN in HGC-27 cells pre-treated with TGF-β RI inhibitor SB431542 (3 h) before TGF-β1 exposure. **F** TGF-β RI expression in HGC-27 cells under attachment and suspension conditions at indicated times. **G** FERMT2 expression in HGC-27 cells pre-treated with SB431542 under attachment and suspension conditions. **H** TGF-β RI expression in sh-Ctrl/sh-FERMT2 HGC-27 cells under suspension conditions for 24 h. **I** Expression of integrins α5 and β1 under suspension and attachment conditions at the indicated times. **J** Expression of integrins α5 and β1 in sh-Ctrl/sh-FERMT2 HGC-27 cells under suspension for 24 h. **K** Schematic of intraperitoneal injection of MKN45 cells with SB431542. **L, M** Effect of shRNA targeting FERMT2 and SB431542 on MKN45 cell peritoneal metastasis, comparing nodule weight and relative weight. **N** Diagram of FERMT2’s role in ECM deposition and cell–cell junctions, enhancing anoikis resistance in GC cells and promoting peritoneal metastasis. (^ns^*p* > 0.05; *p < 0.05; **p < 0.01; ***p < 0.001; ****p < 0.0001)

Using the STRING database, we identified a correlation between FERMT2, TGF-β1, and TGF-βRI (Fig. 5A). In HGC-27 cells, TGF-β1 levels in the supernatant increased under suspension conditions (Fig. 5B). Treatment with 5 ng/ml TGF-β1 for varying times (Fig. 5C) or different concentrations for 24 h (Fig. 5D) upregulated FERMT2 expression (Fig. 5C, D). The TGF-β RI kinase inhibitor SB431542 blocked this effect (Fig. 5E). A suspension-time-dependent increase in TGF-βRI expression was observed, which was inhibited by SB431542 (Fig. 5F, G). Knockdown of FERMT2 reduced TGF-βRI expression under suspension conditions (Fig. 5H).

Integrins α5 and β1 were also upregulated in GC cells under suspension, and FERMT2 knockdown reduced their expression (Fig. 5I, J). Combination treatment with SB431542 and FERMT2 knockdown further inhibited peritoneal tumor formation in MKN45 cells (Fig. 5L, M). These results suggest that the positive feedback loop between TGFβ-1/TGFβ-RI signaling and FERMT2 is crucial in regulating AR.

## Discussion

Cancer stem cells are a small subpopulation within tumors that can self-renew, differentiate, and contribute to tumor initiation, progression, and metastasis [20]. When cancer cells detach from the primary tumor and lose adhesion to the extracellular matrix, stem-like cells can acquire AR, increasing their survival and metastatic potential [21]. In our previous study, we identified that Fermitin family homolog 2 (FERMT2), also known as Kindlin-2, regulates the stemness of GC cells [13]. In the present study, we observed a suspension-time-dependent increase in FERMT2 expression in detached GC cells. Furthermore, overexpression of FERMT2 promoted extracellular fibronectin deposition by stabilizing SOX2, which then binds to the promoter region of FN1. These findings further confirm the role of FERMT2 in modulating stemness in GC cells.

HGC-27, AGS, and MKN45 are widely used gastric cancer cell lines, whereas SNU-1 is a non-adherent, suspension-growing gastric cancer cell line. We selected SNU-1 for its unique growth behavior, which simulates the condition of cancer cells undergoing anoikis due to ECM detachment. Under normal culture conditions, SNU-1 cells form small, loosely aggregated clusters, unlike the larger aggregates seen in adherent gastric cancer cells after detachment. Consequently, FERMT2 expression in SNU-1 cells is relatively low, reflecting their distinct growth pattern. Clinically, during tumor progression, primary tumor cells lose ECM adhesion and enter the peritoneal cavity, lymphatic system, or bloodstream, where they must overcome anoikis to metastasize[22]. Thus, adherent cell lines like HGC-27 and AGS are more representative of these biological behaviors and molecular mechanisms associated with anoikis resistance during metastasis. In contrast, non-adherent lines like SNU-1 are less suitable for studying ECM detachment-induced stress in metastatic progression.

SOX2 is regulated at the protein level in stem cells through the ubiquitin–proteasome system (UPS) [23]. Previous studies show that the methyltransferase Set7 promotes SOX2 monomethylation, enhancing its interaction with WWP2 for ubiquitination, a process inhibited by AKT activation [24]. Additionally, APC and Ube2s mediate SOX2 degradation via K11-linked ubiquitination at Lys123 [25, 26]. These findings highlight that SOX2 is tightly regulated by post-translational modifications (PTMs) to maintain stemness. In this study, we observed that SOX2 expression increased in a time-dependent manner in suspended GC cells compared to adherent cells. FERMT2 overexpression or knockdown also influenced SOX2 levels. We discovered that FERMT2 interacts with SOX2, and FERMT2 knockdown promoted SOX2 ubiquitination and degradation. SOX2, as a transcription factor, binds to the FN1 promoter, as predicted by HumanTFDB. Through CHIP assays and rescue experiments, we identified a FERMT2/SOX2/FN1 regulatory axis. Furthermore, overexpressing SOX2 partially rescued the reduced peritoneal metastasis capability caused by FERMT2 depletion in vivo.

TGF-β1 is secreted by cancer cells as well as stromal cells in the tumor microenvironment, such as CAFs[27, 28]. Tumor-derived TGF-β1 plays a crucial role in maintaining the stem cell properties of cancer cells[29–31], and can also induce epithelial-mesenchymal transition in cancer cells[32, 33], thereby promoting resistance to anoikis and treatments and facilitating cancer metastasis. The androgen receptor was positive in about 40% of GC tissues and was borderline significantly linked to poor progression-free survival. The androgen receptor downregulation reduced GC cell migration, invasion, and suppressed EMT-related pathways [34].In comparison to primary tumors, the androgen receptor and TGFβ pathway transcripts were elevated in patient-derived xenografts (PDXs) and circulating tumor cells (CTCs) from triple-negative breast cancer patients [35]. The androgen receptor binds to regulatory regions of genes involved in the canonical TGFβ signaling pathway, and in prostate cancer, the androgen receptor interacts with androgen response elements (AREs) in the TGFB1 promoter to regulate its transcription [18, 36]. We hypothesize that when GC cells detach from the ECM, cellular stress upregulates the androgen receptor expression, which may trigger the local release or activation of TGF-β1.

In this study, we found that the exogenous addition of human recombinant TGF-β1 increased the expression of FERMT2 and Fibronectin in GC cells. Additionally, knocking down FERMT2 in suspended GC cells decreased the expression of TβRI. Interestingly, when GC cells were cultured in suspension, the expression levels of FERMT2 and TβRI increased in a time-dependent manner compared to the adherent state. However, the addition of SB-431542, a TGF-β receptor kinase inhibitor (TRKI), reversed the time-dependent increase in FERMT2 expression and inhibited the peritoneal metastasis capability of GC cells in vivo.

These experimental results indicate that the positive feedback loop between FEMRT2 and TGF-β receptor I amplify the autocrine signaling of TGF-β1 in response to extracellular matrix detachment. This mechanism plays a crucial role in the progressively increasing expression levels of FERMT2 in the cytoplasm upon cell detachment. Furthermore, FERMT2 interacts with and stabilizes SOX2, reducing its degradation by ubiquitin-mediated proteolysis. This process promotes SOX2 to translocate to the nucleus, where it binds to the promoter region of FN1. Consequently, this interaction promotes the deposition of the extracellular matrix, compensating for the loss of adhesion and facilitating downstream survival signaling. Therefore, our study is the first to identify the role of the TGF-β1/FERMT2/TβRI positive feedback loop specifically in the context of anoikis resistance, rather than merely its involvement in promoting cancer cell proliferation and migration[19].

Additionally, FERMT2 is a key integrin-interacting protein involved in integrin activation [37]. Anoikis, a form of programmed cell death, occurs when cells detach from the extracellular matrix, disrupting integrin signaling [38]. Integrins are critical for transmitting signals between the extracellular environment and the cell interior [39, 40]. We found that integrins α5 and β1 were upregulated in GC cells cultured in suspension. Moreover, FERMT2 knockdown significantly reduced the expression of these integrins.

In conclusion, our study identifies FERMT2 as a key regulator of AR and stemness in GC cells. Through a positive feedback loop involving the TGF-β1/FERMT2/TβRI axis and modulation of SOX2 expression, FERMT2 promotes extracellular fibronectin deposition and increases integrins α5 and β1, enhancing cell–matrix interactions and aggregation. This process boosts AR and facilitates peritoneal metastasis in GC. However, the detailed mechanisms by which FERMT2 inhibits SOX2 ubiquitination remain to be explored.

## Data Availability

The publicly available single-cell sequencing data used in this study can be accessed through the GEO database (GSE210347 and GSE163558). Primary data are available upon request.

## Abbreviations

GC: Gastric cancer
FERMT2: Fermitin family homolog 2
FN: Fibronectin
SOX2: SRY (sex determining region Y)-box 2
TRKI: TGF-β receptor kinase inhibitor
TGF-β1: Transforming growth factor beta
TGFβ-RI: Transforming growth factor beta receptor I
ECM: Extracellular matrix
AR: Anoikis resistance
TMA: Tissue microarray
shRNA: Short hairpin RNA (shRNA)
TCGA: The Cancer Genome Atlas
ChIP: Chromatin immunoprecipitation
CAFS: Cancer-associated fibroblasts (CAFs)
